# Risk factors and service gaps affecting a sustainable work: a qualitative multi-stakeholder analysis in the context of persons with acquired brain injury living in Switzerland

**DOI:** 10.1186/s12913-024-11128-3

**Published:** 2024-06-20

**Authors:** Katarzyna Karcz, Urban Schwegler, Barbara Schiffmann, Monika E. Finger

**Affiliations:** 1 Swiss Paraplegic Research, Guido A. Zäch Strasse 4, Nottwil, 6207 Switzerland; 2 Faculty of Health Sciences and Medicine, University of Lucerne, Lucerne, Switzerland

**Keywords:** Sustainable work, Vocational integration, Qualitative research, Acquired brain injury, Multi-stakeholder analysis

## Abstract

**Introduction:**

Along with the social and economic challenges posed by an aging society, creating work conditions that allow persons to stay healthy and work into old age has become a major task of Western societies. Retaining employment after returning to work is particularly difficult for individuals with a disability, as evidenced by the high rate of premature labor market dropout. Individuals with acquired brain injury (ABI) exemplify this challenge, as it often impairs cognitive, technical, and interpersonal abilities that are crucial in today’s labor market. To effectively support these individuals, vocational integration practitioners require comprehensive knowledge of risk factors for premature labor market dropout and effective strategies for sustainable work.

**Objective:**

This study aimed to identify perceived risk factors and related service gaps regarding sustainable work for people with ABI, as reported by affected individuals, employers, vocational integration professionals, and health professionals.

**Methods:**

Secondary data analysis. Data that was originally collected through seven focus groups and two interviews with persons with ABI, 15 interviews with employers, and 13 interviews with vocational integration and health professionals in the context of the project ‘Sustainable employment’ was re-analysed thematically.

**Results:**

Two major themes of risk factors were identified: (1) person-related factors (including the subthemes: post-ABI impairments; lack of understanding of post-ABI impairments; poor health management) and (2) environment-related factors (including the subthemes: challenges related to the service structure; insufficient knowledge and education about ABI; challenges at the workplace; difficulties in private life). While stakeholders noted the variety of the currently available services, they particularly pointed to the missing long-term monitoring and counseling services for persons with ABI following the initial return-to-work, reflecting a major challenge for sustainable work. An overarching gap related to the fragmentation of the service structure and the lack of case coordination along the working life.

**Conclusions:**

Multiple stakeholders emphasized the importance of empowering individuals, ensuring easy access to professional support, and providing a suitable work environment to address key risk factors and facilitate sustainable work for individuals with ABI. Continuous coaching, long-term monitoring and counseling following return-to-work, were identified as potential strategies to achieve these goals.

**Supplementary Information:**

The online version contains supplementary material available at 10.1186/s12913-024-11128-3.

## Introduction

Along with the socio-economic challenges posed by an aging society, creating work conditions that enable persons to stay healthy and able to work into old age has become a significant task of Western societies [1–5]. For persons with a disability, retaining employment after return-to-work (RTW) represents a particular challenge, as indicated by the high number of premature dropouts from the labor market among this group [6–9]. Therefore, vocational integration services aim to ensure the best possible conditions for supporting sustainable employment throughout a person’s lifespan. A sustainable work situation can be defined as ‘a person-job-workplace match that enables a person to remain healthy and satisfied at work over time, with a work performance meeting the expectations of both the person and the employer’ [10].

Persons with an acquired brain injury (ABI) make an exemplary case for the challenge of sustaining work over time as their health condition strongly affects the ability to perform cognitive, technical, and interpersonal tasks that are increasingly relevant in today’s labor market [11–15]. An acquired brain injury represents an incisive life event resulting in a high monetary and nonmonetary burden for those affected as well as the society [16]. Maintaining gainful employment after ABI is thus important not only for ensuring individuals’ financial self-sufficiency, self-worth, social inclusion as well as quality of life, but also for lowering socio-economic costs and increasing tax revenues [17–19]. ABI reflects a traumatic (i.e., due to an accident) or non-traumatic (i.e., due to a disease such as stroke) damage to the brain that occurs during life and is not related to any degenerative disease such as multiple sclerosis or Alzheimer. While a mild ABI may result in little to no limitations, severe injuries can lead to significant lifelong disability. ABI may involve a wide range of problems, including observable ones such as movement and balance disorders and non-observable ‘silent’ ones like concentration issues, increased fatigability, emotional instability, and planning difficulties [20]. While in some cases functioning may still improve several years after ABI onset [21], it is more common that people with ABI experience a decline in functioning over time [22].

Internationally, there exist approximately 73 million working-age people with ABI, with around 90’000 of them living in Switzerland [23]. RTW rates range from 11% to 85% globally [24–26]. In Switzerland, data on the employment rates for people with ABI is missing, but according to support organizations for people with ABI, a substantial number of those affected struggle to maintain their jobs or drop out from the labor market prematurely [27]. Sustainable employment is particularly hampered by non-visible impairments, that lead employers and co-workers but also people with ABI to overestimate affected workers’ abilities, resulting in a lack of support or the assignment to unsuitable work tasks. Such a work situation can lead to overwork and increased fatigue [28, 29]. Discrepancies between the affected individual and the employer regarding the perception of job performance as well as an unstable work performance can also increase the risk for job loss [30, 31].

In Switzerland, individuals with acute ABI that show significant neurological symptoms typically undergo two to twelve weeks of initial inpatient or outpatient neurorehabilitation. Subsequently, a range of services that are funded by the health, accident or disability insurance supports the recovery as well as the work and community integration of those affected [32]. The Swiss Disability Insurance (IV), a mandatory system funded by wage deductions and federal support, provides a disability pension based on income loss rather than the severity of the injury, which - in case of traumatic brain injuries – is complemented by accident insurance benefits. The IV also covers medical treatments, assistive devices as well as vocational integration services (e.g., vocational counselling, job placement) that are aimed at returning people to the labor market. Fragile Suisse represents the support organization for individuals with ABI in Switzerland and provides help with a particular focus on community integration, including work as well as social counseling with respect to the navigation through the administrative issues in the context of the health and social security system.

To provide optimal support services for persons with ABI, both service providers and payers require conclusive knowledge on risk factors for premature labor market dropout as well as strategies to optimally promote sustainable work. This knowledge may best be generated through a need-driven approach that incorporates the view of the key stakeholders in the vocational integration process. The present study thus aimed to identify risk factors and related service gaps regarding a sustainable work by taking individuals with ABI as an exemplary case and by integrating the view of those affected, their employers, as well as health and vocational integration professionals.

## Methods

To address our research question, we carried out a secondary analysis of data collected in the context of our previous project ‘Sustainable employment’ [33]. Data was collected between May 2019 and July 2020.

### Data source: the ‘Sustainable employment’ project

The original project aimed to identify barriers and facilitators to sustainable employment for individuals with a disability, specifically focusing on those with acquired brain injuries and spinal cord injuries (SCI) [33]. To respond to this study aim, we conducted focus group discussions and semi-structured interviews [34, 35] with individuals with ABI and SCI [36], health and vocational integration professionals [37], as well as employers of persons with ABI and SCI [38]. Based on our finding that the availability and structure of vocational integration services were perceived as a major barrier to employment sustainability, especially for persons with ABI, we decided to conduct an in-depth secondary analysis of the qualitative data, focusing on perceived service gaps and their relation to the identified risk factors for sustainable employment of people with ABI. While the original project analyzed the perspective of all three stakeholder groups separately, the present study merges the perspectives of the stakeholder groups. In the current secondary analysis, the gathered data was re-analyzed thematically [34, 39].

### Participants

The sample included persons with ABI who worked for at least two years after the onset of their injury, employers with experience in employing an affected person as well as health and vocational integration professionals experienced in the topic of ABI. Individuals who were unable to go back to work successfully were not included as the ‘Sustainable employment’ project did not focus on factors affecting RTW but on barriers and facilitators to sustaining employment after having returned to work post-ABI. Employers represented companies of different sizes and sectors (private, public, non-profit). Professionals were expected to be familiar with the spectrum of the existing support services as well as with the administrative and legal regulations of the Swiss health and social security system.

We employed multiple recruitment strategies. Initially, we made announcements in the publications and on the webpage of Fragile Suisse, the support organization for individuals with ABI, their families, and professionals. Additionally, we distributed flyers via health and vocational integration professionals and received recommendations for candidate participants from two vocational rehabilitation departments and the work integration group of the Swiss Association of Rehabilitation. Finally, recruitment was facilitated through personal contacts of authors of this study (MF and BS).

### Data collection

We conducted seven focus groups and two single interviews, including a total of 23 persons with ABI. In addition, we carried out 15 interviews with employers and 13 interviews with professionals. Sample characterstics are presented in Tables 1, 2 and 3. The focus group discussions included three affected persons, lasted between 90 and 120 min and took place in seminar rooms located at different cities in the German-speaking region of Switzerland. We chose focus groups as our primary data collection method for persons with ABI to facilitate discussions and the exchange of experiences among participants with different characteristics. Single interviews were offered to two volunteers who were unable to participate in the focus groups due to their ABI-related limitations. These interviews were conducted at participants’ homes and took between 45 and 90 min. Interviews with employers and professionals lasted between 30 and 70 min and were conducted at their workplace, except two being carried out at the Swiss Paraplegic Research Institute in Nottwil and three that were conducted online via Zoom. Interviews were chosen as data collection method for employers and professionals due to time constraints, organizational barriers and ethical issues (the necessity to maintain anonymity of persons with ABI). The focus groups and the interviews followed the same procedure of discussing facilitators and barriers for sustainable work, service gaps and risk factors for labor market dropout. Detailed information on the data collection is published elsewhere [36–38]. The guidelines of the focus groups and the interviews are provided in the supplementary material (S1-S4).

**Table 1:** Characteristics of study participants with ABI

	Age (years)	Gender	Employment status	Current job title (assigned to ISCO-08* major groups)	Time since injury (years)
1	30–39	male	employed	Clerical support workers	10–19
2	30–39	male	employed	Clerical support workers	< 10
3	30–39	female	employed	Professionals	< 10
4	30–39	male	employed	Service and sales workers	< 10
5	30–39	female	employed	Professionals	< 10
6	30–39	male	employed	Professionals	10–19
7	40–49	female	employed	Clerical support workers	10–19
8	40–49	male	unemployed	Professionals	10–19
9	40–49	female	employed	Service and sales workers	10–19
10	40–49	female	employed	Clerical support workers	< 10
11	40–49	female	employed	Technicians and associate professionals	20–29
12	40–49	female	unemployed	Agricultural worker	10–19
13	40–49	female	employed	Clerical support workers	10–19
14	40–49	male	unemployed	Service and sales workers	< 10
15	40–49	male	employed	Technicians and associate professionals	< 10
16	50–59	male	employed	Craft and related trades workers	20–29
17	50–59	male	employed	Service and sales workers	10–19
18	50–59	female	retired	Service and sales workers	< 10
19	50–59	female	employed	Professionals	< 10
20	50–59	female	employed	Service and sales workers	20–29
21	50–59	female	employed	Managers	10–19
22	50–59	male	employed	Clerical support workers	20–29
23	60+	male	employed	Professionals	10–19

*ISCO-08 International Standard Classification of Occupations by the International Labor Organization (ILO) [40]

**Table 2:** Characteristics of employers participating in the study

	Company type	Company size	Direct superior?	Person with a disability employed by company before injury
1	Foundation	Small	Yes	No
2	Private enterprise	Medium	No/HR	Yes
3	Public sector	Small	Yes	No
4	Private enterprise	Medium	No/HR	Varying
5	Private enterprise	Small	No	No
6	Public sector	Large	Yes	Yes
7	Public sector	Medium	Yes	Yes
8	Public sector	Large	Yes	Yes
9	Public sector	Large	Yes	No
10	Private enterprise	Medium	Yes	Yes
11	Foundation	Small	Yes	Varying
12	Private enterprise	Small	Yes	Yes
13	Foundation	Large	No	No

Company Size: Micro: 1 to 9 employees; small: 10 to 49 employees; medium: 50 to 249 employees; large: 250 and more employees (Swiss Federal Statistical Office, Small and Medium-sized Enterprises, https://www.bfs.admin.ch/bfs/de/home/statistiken/industrie-dienstleistungen/unternehmen-beschaeftigte/wirtschaftsstruktur-unternehmen/kmu.html)

**Table 3:** Characteristics of professionals participating in the study

	Years of work experience	Main phase of engagement along work continuum	Profession and work experience
1	10	Integration	Vocational specialist In- and outpatient rehabilitation
2	8	Integration	Vocational specialist Outpatient rehabilitation
3	9	Integration and long-term	Consultant Patient organization, consulting
4	10	Integration	Job coach, NGO
5	> 5	Integration and long-term	Patient advocate
6	34	Integration and long-term	Physician, neurologist
7	24	Integration and long-term	Social Counselor, NGO
8	8	Integration	Job coach, self-employed
9	15	Integration	Job coach, self-employed
10	10	Integration	Vocational specialist In- and outpatient rehabilitation
11	8	Integration	Case manager, disability insurance
13	14	Integration and long-term	Administrator Blended counselling, disability insurance
14	16	Integration and long-term	Vocational specialist, In- and outpatient rehabilitation
15	12	Integration and long-term	Case manager, accident insurance

Focus groups and interviews were audio recorded, transcribed verbatim and anonymized. MF (a physiotherapist experienced in qualitative research) and BS (a sociologist experienced in qualitative research) conducted the focus group discussions and the interviews. The first author (KK, a sociologist and psychologist with experience in qualitative research) participated in all focus groups as an assistant and note-taker.

### Data analysis

We conducted a thematic analysis according to Braun & Clarke [34, 39] with an inductive coding throughout the process. Data were coded by KK using the software MaxQDA. Additionally, MF independently coded one interview from each participant group. KK and MF compared and discussed then the coding schemes until agreement was achieved. We operationalized risk factors as ‘aspects associated with an increased risk of unsustainable work’ and service gaps ‘as differences between the actual support provision and the participants’ support needs’. Risk factors were coded inductively (e.g., rapid fatigability, headache) and then grouped into subthemes of similar factors (e.g., post-ABI impairments). The subthemes formed the two overarching themes, i.e., person-related and environment-related factors. Identified service gaps were first coded inductively and then assigned to the related risk factors. The coding scheme and the assignment of the subthemes were extensively discussed among all authors throughout the coding process.

## Results

 In the following, we describe the service gaps reported by our participants in relation to the perceived risk factors for sustainable work. Sub-themes are grouped into the overarching themes person-related and environment-related factors (see Table 4).

**Table 4:** Risk factors and corresponding service needs

Overarching theme	Themes and sub-themes of risk factors	Service needs during first rehabilitation, vocational rehabilitation, and work integration	Reasons for a service gap*			Service needs in the long term	Reasons for a service gap*		
			Aw	Acc	Ex		Aw	Acc	Ex
Person-related factors	**Post-ABI impairments**	(Neuro)-Psychological counselling.	X	X		Access to long-term counselling on request.		X	X
		Coaching, (neuro)- psychotherapy.							
		Confronting deficits during vocational rehabilitation, e.g., in context of supported employment (by work professional).							
		Strengthening patient empowerment during vocational rehabilitation and integration.							
	**Lack of person's understanding and acceptance of post-ABI impairments and its consequences for work**	(Neuro)-Psychological counselling.	X			Coaching, psychotherapy on request.	X		
		Coaching, (neuro)- psychotherapy.				Mediation involving workplace, and/or insurances.			
	**Poor health-management**	Confronting deficits during vocational rehabilitation, e.g., in context of supported employment (by work professional).							
		Strengthening patient empowerment during vocational rehabilitation and integration.							
Environment-related factors	**Challenges related to the service structure**								
	• Fragmentation of services and lack of case management across the work life	One central organization, supervising case management cross the lifespan (IV).			X	One central organization, supervising case management cross the lifespan (IV).			X
	• Fixed timelines in vocational integration process	Recovery dependent procedures instead of fixed time schedules, e.g., time-point of final assessment, monitoring of health state and time point of case closure at insurance level.			X	Not applicable.			
	• Confusing and complicated administrative processes for persons with ABI	Standardized and client friendly administrative processes (insurances and social system).			X	Standardized and client friendly administrative processes (insurances and social system).			X
	• Lack of common ABI special guidelines and inadequate funding: - patients' organizations financed partly by fundraising, - type and duration of intervention must be negotiated with a payer, - no legal basis for beyond standard VR interventions targeting ABI specific needs.	National standards for clinical assessment, linked to service provision should be agreed on and encouraged.			X	Easy access to specialists (neuropsychologist, psychiatrist, job coach).			X
		To avoid missing an ABI diagnosis, a multidisciplinary assessment involving neurologic specialists should be performed as soon as there is evidence of brain injury.				Easy access to reassessment of disability benefit in case of change in health status and work ability by insurance, to support work adjustments (workload reduction).			
		Beyond standard VR interventions agreed upon and targeted to specific ABI needs.							
Environment-related factors	**Insufficient knowledge and education about ABI**								
	• Limited knowledge about existing services offer among work, health and insurance professionals	Further training. Coordination of services along work continuum with focus on interfaces between services and environmental changes.	X	X	X	Further training. Coordination of services along work continuum with focus on interfaces between services and environmental changes.	X	X	X
	• Lack of knowledge about post-ABI impairments among HR professionals, supervisors, and co-workers	Further training.	X	X	X	Further training.	X	X	X
		Professional advisory: - mediations in conflicts at the workplace, - sensitization of co-workers and supervisor.				Professional advisory on request: - mediations in conflicts at the workplace, - sensitization of co-workers and supervisor.			
		Helpline to get quick support/answer/contact to find advisory.				Helpline to get quick support/answer/contact to find advisory.			
Environment-related factors	**Workplace challenges**								
	• Poor person-job match and overburden	Coaching with special consideration for job-match assessment.	X	X		Coaching on request.	X	X	
	• Workplace restructuring: - change of supervisor, - change of work tasks, - changes at the workplace.	Change management policy in a company (sharing of information and procedures with new supervisors/team members).		X	X	Change management policy in a company (sharing of information and procedures with new supervisors/team members).		X	X
		Professional advisory: - coaching in case of work-related problems, - mediations in conflicts at the workplace, - sensitization of co-workers and supervisor.				Access to professional advisory if needed: - coaching in case of work-related problems, - mediations in conflicts at the workplace, - sensitization of co-workers and supervisor.			
		Helpline to get quick support/answer/contact to find advisory.				Helpline to get quick support/answer/contact to find advisory.			
	• Lack of open communication and continuous expectation management	Coaching.	X	X		Coaching on request.	X	X	
	• Dealing with insurance procedures	Administrative support for employer/affected person regarding insurance and other procedures concerning work.	X	X		Administrative support for employer/affected person regarding insurance and other procedures on request.	X	X	
		Helpline to get quick support/answer/contact to find advisory.				Helpline to get quick support/answer/contact to find advisory.			
Environment-related factors	**Difficulties in private life**								
	• Family conflicts	Support/counselling for relatives, family therapy.			X	Support/counselling for relatives, family therapy on request.			X
	• Missing support for family members	Respite care, health assistance			X	Respite care, health assistance.			X

*Aw – awareness; Acc – accessibility, Ex – existence

### Person-related risk factors and service gaps

#### Post-ABI impairments

Individuals emphasized the negative impact of cognitive post-ABI impairments on their work performance, while employers highlighted the unstable health and the unpredictability of work performance, leading to difficulties in the work organization. A common problem represent the affected individuals’ inability to accurately assess and predict their own work capabilities, resulting in potential errors or unmet deadlines. All stakeholder groups agreed that a close monitoring by a vocational integration specialist during the return-to-work phase is essential, including professional coaching to adjust job demands based on the individual’s recovery state. Professionals pointed to the various work counseling services, typically funded by the IV, but noted that the accessibility and timing of these services are often unstructured and depend on whether vocational measures are part of the rehabilitation process or initiated later by requests of those affected, their general practitioner or a caregiver.

#### Lack of understanding of post-ABI impairments and poor health management

All stakeholder groups recognize that the poor understanding of ABI-related impairments and the inability to recognize body signals, like fatigue, jeopardizes stable employment. Without this awareness, it’s hard for those affected to develop a disability-friendly life and work style. Stakeholders emphasized the need for vocational integration specialists and psychologists that teach effective self-management strategies already during rehabilitation to empower persons with ABI and support in becoming experts in their own disability. However, professionals reported that medical staff’s tight schedules pose challenges to providing this education and care effectively during the medically-oriented initial rehabilitation.

Persons with ABI and health professionals consider ongoing personal coaching from neuropsychologists, occupational therapists, or vocational integration specialists as crucial for managing post-ABI challenges and adapting to new career paths. While such support is typically provided in neurorehabilitation, the awareness and availability of such services are lower among those with mild to moderate injuries who are discharged early from rehabilitation. Professionals also highlighted the need for extended neuropsychological coaching beyond the vocational integration phase to promote sustainable employment. Yet, accessing these services is difficult due to complex procedures, long waiting times for approvals, and limited resources of patient organizations.

### Environment-related risk factors and service gaps

#### Challenges related to the service structure

##### Fragmentation of services

Service fragmentation was identified as a critical gap by all stakeholder groups, noting a poor coordination across different phases of working life. The RTW period was especially emphasized, with various services offered by governmental, non-governmental, inpatient, outpatient, and private institutions to support disability onset. However, stakeholders highlighted significant challenges in the coordination and accessibility of these services. Additionally, they pointed to regional differences in service regulations and financing, influenced by factors like workplace location, employer’s insurance, and availability of private insurance options.

##### Lack of case management across the work life

Participants reported a significant gap in case management across the work life of individuals with ABI, noting a lack of long-term coordination between various service settings such as inpatient, outpatient, and long-term care. They highlighted the absence of a dedicated person or institution to guide through the system procedures, leaving individuals with ABI to navigate their post-discharge care pathway independently.

Furthermore, all groups emphasized the need for long-term monitoring that integrates medical and work-related issues to address potential functional declines that could affect work performance. Despite this need, IV cases typically close after two years, assuming stabilization. Although the person or their employer can still apply for support even after integration has been completed, long waiting times for a counselling appointment and a high administrative burden often discourage both from contacting the IV. Participants pointed out that in these situations, the support of patient organizations is crucial for persons with ABI, their families and their employers. Patient organizations may take on case coordination in terms of acting as an intermediary between affected persons and the insurances or finding suitable providers (specialists, coaches, shift workers, etc.). When a patient organization was involved, its support was largely successful according to the study participants.

##### Fixed timelines in the vocational integration process

Participants criticized the rigidity of the vocational integration process for individuals with ABI, including the fixed timing of work capacity assessments used to determine disability pensions that often occure before individuals reached a stable recovery state. Shifting work capacity assessments until stable stage could lead to a more effective and sustainable RTW processes. In addition, persons with ABI would like more flexibility in their daily or weekly workload so as not to be overburdened when their work capacitiy fluctuates for health reasons – employers on the other hand pointed out that they need employees with a workload that is as reliable as possible. Although reassessment of work capacity is possible when it fluctuates due to health issues, it typically happens late, under duress, and involves lengthy administrative procedures. Professionals suggest that allowing for timely adjustments of workloads would prevent overburdening, and benefit the individual, the workplace as well as the social system both economically and health-wise.

##### Confusing and complicated administrative processes for persons with ABI

Although participants shared both, negative and positive examples of working with insurance companies, all stakeholders found dealing with administrative procedures or regular disability benefits revisions to be stressful and time-consuming. According to employers and professionals, the stress and frustration experienced by persons with ABI in dealing with insurance policies leads to further decline in their work performance.

##### Lack of common ABI-specific guidelines and inadequate funding

The absence of established guidelines, leads to insufficient funding due to a lack of legal support. Medical professionals highlight that this results in insufficient funding for adequately long inpatient stays in emergency, acute care, and initial rehabilitation, often causing missing or incomplete ABI diagnoses and inadequate vocational interventions. They urge the creation of standardized guidelines to support financial claims and recommend an interdisciplinary team, including neurologists or neuropsychologists, for diagnosing suspected ABI. Limited resources also restrict follow-up assessments to properly evaluate recovery post-ABI. Additionally, the lack of guidelines which would provide a basis for financial claims impedes also long-term support, such as job coaching for affected individuals and employers. Despite patient and employer organizations stepping in to some extent, fundraising and lack of resources create a barrier to meet the support needs.

#### Insufficient knowledge and education about ABI

Insufficient knowledge about ABI was identified as a significant gap in two areas. First, the discrepancies in professionals’ knowledge on ABI, its procedures, and its impact on daily life leads to inconsistent support, depending heavily on the competence and network of the case manager or specialist involved.

Second, professionals and affected persons reported a lack of knowledge about ABI and its impact on work and daily life among employers and co-workers, resulting in a lack of understanding of persons’ needs in relation to ABI-specific work adjustments. To address this, training HR staff or designating a person within the company to learn how to best support persons with ABI as well as their supervisors and co-workers could be a promising intervention target.

#### Workplace challenges

##### Poor person-job match

One of the key challenges to maintain the work over time is to establish a good person-job match, which broadly refers to the fit between persons’ abilities, needs, and interests and the requirements and characteristics of their jobs [41]. As reported by all stakeholder groups, person-job mismatches were often related to too many or too difficult job tasks. In the long run, such situation can lead to overwork and premature dropout. To prevent these issues, it’s recommended that the final assessment of an employee’s work capacity and support needs should be conducted only after reaching a stable post-injury state, with regularly follow-ups regarding person-job match by both the employee and supervisor.

##### Workplace restructuring

All stakeholder groups perceived changes in the work situation as a risk factor due to the high level of adjustment required from those affected. Moreover, when the work environment changes, arrangements that have been put in place to support the employee are likely to be discontinued. Additional professional advice, such as coaching or mediation, should be offered on request for challenging cases. However, the availability and funding of such services is limited. Employers expressed a wish for a single point of contact or helpline where they could get immediate support regarding key issues or access to a specialist if needed.

#### Difficulties in private life

Professionals and employers frequently cited personal life issues as a significant risk factor for individuals with ABI. Family conflicts or changes in support networks can have a more negative impact than before the onset of ABI, draining personal resources and reducing work capabilities. To mitigate this, professionals recommend providing psychological support to family members and educating them about ABI consequences and available supports, such as through a helpline. At present no such services exist apart from support from patients organizations.

## Discussion

Based on a multi-stakeholder approach, our study identified service needs related to the sustainable employment of persons with ABI by linking existing services and perceived service gaps with main risk factors for sustainable work. We identified several person-related (e.g., lack of understanding of post-ABI impairments) and environment-related risk factors (e.g., fragmentation of services; workplace restructuring) associated with different stages of vocational integration (rehabilitation, work integration, and long-term work-related support).

Perceived service gaps were found to be highly interdependent and mainly related to accessibility, funding and availability of services. Service quality was mainly an issue in terms of poor professional commitment and ABI-related knowledge.

### Person-related factors

Our results point to the need of services that enhance the understanding of ABI impacts on daily life, that empower individuals to assess their abilities, and that teach self-management of health. While self-management of disability in daily life is recognized as important [42–49], rehabilitation and vocational integration services typically prioritize physical and vocational skills over person-centered skills, despite evidence supporting the importance of empowerment early on [24–28]. Most participants also reported difficulty accessing services focused on person-centered skills in outpatient or vocational settings after insurance claim closed, particularly for injuries deemed ‘minor’. This reflects a lack of recognition of ABI as a chronic condition that can improve or worsen over time [50–53]. Interdisciplinary guidelines and ABI-related training for professionals could improve the focus on person-centered skills in medical and vocational programs.

### Environment-related factors

Our results suggest that an ideal setting for work integration would be on-the-job mentoring by a vocational counsellor who coordinates the patient’s skills and needs with the employer’s possibilities and expectations and the funding (insurance). Since there is no legal basis in Switzerland that obliges an employer to continue to employ a worker with reduced work capacity, a close cooperation with the employer is crucial for successful RTW and long-term employment. Participants of our study highlighted that the possibility of a partial pension can help persons with a disability and their employers to find a mutually agreed person-job match which is a central aspect of vocational integration [54, 55].

When it comes to finding a new employment, injured workers are largely left to their own devices, due to limited support from the IV or public employment services, exacerbated by a lack of trained staff to assist those with invisible impairments like ABI [56]. Our findings imply that employment offices establish specialized units with medically trained staff to improve support for these individuals.

After RTW, both employer and employee must continually assess and adjust the work situation as needed. However, over 90% of companies in Switzerland are small and medium-sized enterprises [57] which do not have trained staff and cannot rely on their own resources. Therefore, when problems arise in terms of long-term health management, an official service with easy and widely known access to counseling and timely vocational support measures would be important to preserve sustainable employment. This could be managed by IV or through support organizations like Fragile Suisse [27, 58].

Finally, the impact of ABI extends beyond the individual to affect caregivers and family members, who face significant unacknowledged burdens in terms of direct care, taking on household tasks, reduced social contact and financial losses due to reduced working hours. In Switzerland, this support goes widely unnoticed, although continuous overburden may lead to severe psychological, physical and social problems [27]. Enhancing caregiver support through standardized assessments and providing psychological and psychosocial support could mitigate the profound challenges they face.

### The big picture

Despite Switzerland disposing one of the best health and social security systems globally [59], it suffers from significant fragmentation in financing and service provision across systems, institutions, providers, and settings. As visible in our results, different legal foundations, unclear responsibilities and poorly coordinated processes lead to patients receiving inadequate care despite the comprehensive range of services provided.

Recent legal revisions aim to improve work integration measures, with regional efforts enhancing coordination with local job offices. However, patients often hesitate to engage with administrative procedures, as navigating their complexities can be overwhelming. Fragile Suisse’s pilot projects [58], providing comprehensive support from medical incidents to social or professional reintegration, are well-received but under-resourced. These projects suggest that a Switzerland-wide formal case coordination approach could significantly improve vocational reintegration and sustainable work for people with ABI. Adopting a structured service pathway could enhance service overview, enable performance evaluation, and make the health and social systems more cost-effective. The Swiss Paraplegic Foundation’s model [60] for spinal paralysis could serve as a blueprint for managing ABI and other chronic conditions.

### Strengths and limitations

A strength of our study was the inclusion of a broad range of stakeholders who are directly confronted with service needs and gaps across the work-lifespan of persons with ABI - from initial rehabilitation through vocational rehabilitation, sustained work, and retirement. Using this approach, we were able to identify individual service needs at the micro level and discuss overarching challenges at the meso and macro level that affect the continuum of service provision.

A limitation of this study is the analysis of previously collected data. Although, participants discussed services and service gaps for sustainable employment in the original interviews, services were not the main focus of the primary study. Additionally, the majority of persons with ABI were employed, therefore some risk factors could have been missed.

### Practical, policy and research implications

Existing research underscores the importance of work for the psychosocial adjustment and health of individuals with ABI, with RTW contributing to higher self-esteem, life satisfaction, and economic independence [61, 62]. Our study advocates for a policy shift towards funding coordinated long-term, participation-oriented interventions as part of a comprehensive service provision along the continuum of care.

Addressing fragmentation of services through the coordination of settings and measures would optimize not only the support for persons with ABI but also for people with other disability or long-term conditions and their sustainable work [63, 64]. Establishing national guidelines developed and agreed upon by an interdisciplinary group of stakeholders (service providers, insurances, policy makers and affected persons as well as their employers) with the use of an integrated knowledge translation approach [65–67], and developing a framework to monitor service effectiveness are critical for optimizing interventions. Additionally, professional education and quality measures need enhancement.

Currently, Switzerland lacks a national database on ABI, service provision, and employment status. Therefore, cohort studies that track services, health, social, and RTW outcomes are essential for creating effective strategies. Adopting a mixed-methods approach that combines longitudinal labor market data with qualitative research will help to identify the most effective support strategies for sustainable work participation among those with ABI.

## Conclusions

Our study highlights service gaps in vocational integration and long-term employment for persons with ABI in Switzerland, linking these gaps to existing risk factors for sustainable work. A key finding is the need for better integrated care and streamlined service pathways focused on sustainable vocational integration, including services that support person-centered skills and case coordination throughout the working life. The development of national guidelines for selecting and timing interventions, along with implementing a comprehensive monitoring system, could enhance the evaluation and optimization of intervention quality and service efficacy.

### Supplementary Information

Supplementary Material 1.

Supplementary Material 2.

Supplementary Material 3.

Supplementary Material 4.

## Data Availability

The datasets generated and/or analysed during the current study are not publicly available in order to protect the anonymity of participants but are available from the corresponding author on reasonable request.
