# Supplementary material for: Risk factors and service gaps affecting a sustainable work: a qualitative multi-stakeholder analysis in the context of persons with acquired brain injury living in Switzerland
Source: BMC Health Serv Res. 2024 Jun 20;24:753. doi: 10.1186/s12913-024-11128-3 (PMC11188514; doi:10.1186/s12913-024-11128-3)
Supplement: Supplementary file 1 — Supplementary Material 1. [file 12913_2024_11128_MOESM1_ESM.docx]

**Focus Group Guidelines**

**Preamble** The group discussion involves 3 to 6 participants who may not necessarily know each other. All participants have an ABI (Acquired Brain Injury) and have worked continuously for at least 2 years post-ABI. Participants may differ significantly in gender, age, lesion height, type of lesion, time since the incident, and work experience (maximum variation sampling).

The group discussion aims to capture the experiences of the participants, thereby gaining insights into the topic of "long-term satisfying employment with ABI in Switzerland".

A guideline has been prepared in advance, selecting and arranging questions to stimulate yet structure the flow of the conversation.

The discussion, including the introduction of topics, is led by a moderator tailored to the target audience. The discussion is digitally recorded. A minute-taker notes the sequence of the conversation, including seating arrangements, transitions to new questions, and participant exchanges. These notes, which make as many concrete references to the discussion as possible, aid the researchers in accurately transcribing the electronically recorded discussion rounds and in subsequent analysis. It is important to ensure that participants are anonymously marked (with age and gender).

**Agenda**

- **Preparation**

**Invitation / Welcome:** Participants should arrive 15 minutes before the start to clarify administrative questions (informed consent) beforehand. This also ensures that the group can start on time. Welcoming the participants, establishing contact.

**Room Setup:** Arrange tables and chairs in a circle around the tables, set up microphones, place a clearly visible flipchart. Choose a wall for sticky notes, distribute pens, name tags, green and red sticky cards among the participants, and place drinks on the tables.

**Materials:** Audio recorder, meeting protocol, declaration of consent, questionnaire, flip chart with questions, blank name tags, sticky cards, pens for sticky cards and questionnaire, drinks, cups, envelopes for expenses, address block for collecting addresses.

- **Introduction**

Brief introduction by the moderators. Depending on the assessment, the moderators may ask if participants prefer to use informal language (Du).

Thank participants for their willingness to participate in the study.

Introduction to the topic: the overall project's goal and the focus group's goal. Information to participants: There is no right or wrong. It's important that everyone gets a chance to speak. Therefore, the moderator might interrupt participants at times.

- **Assessing the Evidence from a Personal Perspective**

Introduction round: (Name, age, current occupation, previous occupation, motivation for participating in the study). Work experience with ABI / "Working with Spinal Cord Injury"?

Brainstorming:

- - What is important for "you" to be able to work satisfactorily with spinal cord injury?
  - What are the biggest challenges or reasons why you are not employed?

Participants are asked to write their thoughts on sticky notes. Supportive thoughts should be written on green notes, and challenges or difficulties on red notes.

Each thought on a note - keyword.

Possible support: Think about personal needs and abilities, the environment, the workplace, social and legal frameworks, and people around you.

Offer support if necessary (e.g., hand function).

Gathering and discussing: Collect the sticky notes and stick them on a flipchart. Sort the cards. Participants comment on their own cards. The moderator asks other participants if the mentioned issues are also important to them.

Presentation of the results from the literature review / Complementing the participants' results with those from the literature review (sticky cards: thematic circles).

Questions:

- - What topics that we found in the literature review are or were important/supportive in enabling or simplifying your work?
  - What topics:
    - Complicate your work?
    - Led to quitting work?
    - Prevent you from starting a new activity?
  - Possibly follow up: Concrete situations and experiences.
- **Supplementing Presented Factors**

Is there anything else missing now?

- **Evaluating the Factors**

You will now receive 3 green and 3 red dots. Please mark the areas with green dots that help you the most in working satisfactorily. Mark the most significant challenges/hindrances with red dots. Offer support if necessary (e.g., hand function). Does the evaluation present a uniform picture? Discuss the distribution together. Reflect on the evaluation: Why are there clusters? What is particularly helpful or hindering? Why are the evaluations very different?

What are your "red flags" or warning signs of a looming long absence of the employee / difficult work situation that you pay attention to or have experienced?

- **Support Needs**

What kind of offers or support would you personally wish for:

- - to ensure that you can stay at your job (physically, mentally, work-related measures)?
  - to improve your current work situation? or
  - to enable you to return to a satisfying job?
- **Conclusion of the Focus Group and Questionnaire**

Are there any additions or recommendations you would like to give us? Please now fill out the questionnaire. We are happy to assist if anything is unclear.

- **Farewell**

Collect addresses if participants wish to be informed about the results. Collect addresses from employers / professionals. Pay travel expenses. Personal thanks and farewell.
