# Supplementary material for: Risk factors and service gaps affecting a sustainable work: a qualitative multi-stakeholder analysis in the context of persons with acquired brain injury living in Switzerland
Source: BMC Health Serv Res. 2024 Jun 20;24:753. doi: 10.1186/s12913-024-11128-3 (PMC11188514; doi:10.1186/s12913-024-11128-3)
Supplement: Supplementary file 2 — Supplementary Material 2. [file 12913_2024_11128_MOESM2_ESM.docx]

**Guidelines for Individual Interviews with Affected Individuals**

**Agenda**

- **0. Preparation**

**Materials:** Audio recorder, interview protocol, declaration of consent, questionnaire, topic cards

- **1. Introduction (5 minutes)**

Greet and thank for willingness to participate. Introduce (SPF / Project staff, study goal, interview execution (topic, duration)). Explain data protection and anonymity. (If necessary) Sign the consent form. Express interest in their perspective; open and spontaneous answers; there is no right or wrong. Introduce: occupation, workplace, activity, how long have they been there?

Declaration of consent.

- **2. Assessing the Evidence from a Personal Perspective (20 minutes)**

What is important for you to be able to work satisfactorily with a spinal cord injury?

What are the biggest challenges for you, or why are you not employed?

Possibly support: Think about yourself, needs, and abilities, the environment, the work world, social and legal conditions, and people around you.

Presentation of the results from the literature review / Complementing the results by the results of the literature review.

Which topics found in the literature review are or were important/supportive in enabling or simplifying your work activity?

Which topics:

- - Complicate your work
  - Led to quitting work
  - Prevent you from starting a new activity?

Possibly follow-up: Concrete situations and experiences.

The interviewer notes topics on cards or lets the affected person do it.

- **3. Supplementing the Presented Factors (5 minutes)**

Is there anything else missing now?

- **4. Evaluating the Factors (10 minutes)**

What three aspects help you the most to work satisfactorily?

What are the three most important challenges/hindrances?

What are the "red flags" or warning signs for a looming long absence of the employee / difficult work situation that you pay attention to or have experienced?

- **5. Support Needs (5 minutes)**

What kind of offers or support would you personally wish for:

- - to ensure that you can stay at your job (physical, mental, work-related measures)?
  - to improve your current work situation? or
  - to enable a return to a satisfying work?
- **6. Conclusion of the Interviews and Questionnaire (15 minutes)**

Are there any additions or recommendations you would like to give us?

Please now fill out the questionnaire. We are happy to assist if anything is unclear.

- **7. Farewell (5 minutes)**

Collect addresses from employers / professionals.

Personal thanks and farewell.

Top of Form
