# Supplementary material for: Risk factors and service gaps affecting a sustainable work: a qualitative multi-stakeholder analysis in the context of persons with acquired brain injury living in Switzerland
Source: BMC Health Serv Res. 2024 Jun 20;24:753. doi: 10.1186/s12913-024-11128-3 (PMC11188514; doi:10.1186/s12913-024-11128-3)
Supplement: Supplementary file 3 — Supplementary Material 3. [file 12913_2024_11128_MOESM3_ESM.docx]

**Guideline for Professionals: Work, Health, Social Insurance**

**Preamble** Individual interviews are conducted with professionals at their workplace or a mutually selected location. Professionals are defined as health or employment experts who have supported, trained, or coached individuals with spinal cord or brain injuries in professional integration and possibly later at their regular workplace. The work of the professional may also include mediating between the employer/workplace and the coachee.

The interview aims to capture the experiences of the participants and thus contribute to knowledge acquisition on the topic of "long-term satisfactory work with a spinal cord or brain injury in Switzerland."

In a guideline, questions were selected and arranged in advance to stimulate the conversation flow while simultaneously structuring it.

The discussion, including the introduction of topics, is led by a trained interviewer. The conversation is digitally recorded. The interviewer also notes key points of the conversation. These notes help researchers structure the interview and later accurately transcribe the electronically recorded conversation and during subsequent analysis.

It is important to ensure that participants are anonymized in the notes and subsequent transcript (by age and gender).

**Study Question/Goal:** What helps or contributes to individuals with physical or cognitive disabilities having a healthy and satisfying work life? Focusing on individuals with spinal cord or brain injuries. Identifying factors and underlying mechanisms that help the target group contribute to a healthy and satisfying work life.

**Agenda**

- **Materials:**
  - Audio recorder
  - Interview protocol / Notepad
  - Topic circle diagram template
  - Consent form
- **1. Introduction (5 minutes)**
  - Greet and thank for willingness to participate.
  - Introduce (SPF / Project employee, study goal, interview execution (topic, duration)).
  - Inform about data protection and anonymity.
  - (If necessary) Sign the consent form.
  - Express interest in their perspective; open and spontaneous answers; there is no right or wrong.
- **2. Positive Experiences / Challenges (15 minutes)**
  - Introduce: Profession, function in the company, how long there?
  - What experience do you have with employees with a spinal cord or brain injury?
  - From your perspective, what is necessary for a person with a spinal cord or brain injury to be successful in employment?
  - Support with topic circle diagram: Are there any other important themes in these areas for positive collaboration? For example, the person themselves, work performance, work environment, social system (themes from science).
  - After receiving additional info: From your perspective, are there additional success factors that lead to the long-term integration of an employee with a spinal cord or brain injury at a workplace?
- **3. Challenges (15 minutes)**
  - As a professional, what are the biggest challenges for you?
  - Support with topic circle diagram: Are there challenges in these areas?
  - What are/were the biggest challenges in working with individuals with a spinal cord or brain injury regarding work integration?
  - What are the "red flags" or warning signs for a potential long-term absence of the employee / difficult work situation?
- **4. Support Needs / Offer (10 minutes)**
  - What (which measures) can you offer to support the affected individual or the employer?
  - What other support options are you aware of/use?
  - Deepen: By whom, how, type of support, expectations from the support?
  - From your perspective, what additional offers/measures/legal frameworks are needed?
  - How can a long-term win-win situation for employers and employees be achieved?
- **5. Closing the Interview (45 minutes)**
  - From your perspective, are there any additions or recommendations you would like to give us/something that we should not forget?
  - Thanks and farewell.
