# Supplementary material for: Risk factors and service gaps affecting a sustainable work: a qualitative multi-stakeholder analysis in the context of persons with acquired brain injury living in Switzerland
Source: BMC Health Serv Res. 2024 Jun 20;24:753. doi: 10.1186/s12913-024-11128-3 (PMC11188514; doi:10.1186/s12913-024-11128-3)
Supplement: Supplementary file 4 — Supplementary Material 4. [file 12913_2024_11128_MOESM4_ESM.docx]

**Employer Interview Guideline**

**Preamble** The interview is conducted individually with employer representatives, direct supervisors, or individuals from the Human Resources department who have managed individuals with spinal cord injuries or brain injuries in their professional integration and regular employment.

The interview aims to gather experiences from the participants to gain insights into the topic of "long-term satisfied working with a spinal cord injury or brain injury in Switzerland."

A guideline was prepared in advance, selecting and arranging questions to stimulate the conversation while structuring it simultaneously. The conversation, including the introduction of topics, is led by a trained interviewer. The discussion is digitally recorded, and the interviewer also notes key discussion points.

These notes aid researchers in structuring the interview, accurately transcribing the electronically recorded conversation, and evaluating it later. It is crucial to ensure that participants are anonymized in the notes and subsequent transcript (by age and gender).

**Study Question/Goal:** What helps or contributes to individuals with physical or cognitive disabilities having a healthy and satisfied working life? Focusing on individuals with spinal cord or brain injuries. Identifying factors and mechanisms behind helping the target group contribute to a healthy and satisfied working life.

**Agenda**

- **Materials:**
  - Audio recorder
  - Interview protocol / Notepad
  - Topic circle diagram template
  - Consent form
- **1. Introduction (5 minutes)**
  - Welcome and thank for willingness to participate.
  - Introduce (SPF / Project staff member, study goal, interview process (topic, duration)).
  - Inform about data protection and anonymity.
  - (If necessary) Sign the consent form.
  - Express interest in their perspective; open and spontaneous answers; there is no right or wrong.
  - Introduce: Function in the company, how long there?
- **2. Positive Experiences / Challenges (15 minutes)**
  - What experience do you have with employees with a spinal cord injury or brain injury?
  - What has been positive for you in collaboration with a person with a spinal cord or brain injury?
  - Support with topic circle diagram: Are there any important themes in these areas for positive collaboration? For example, the person themselves, work performance, work environment, social system (themes from science).
  - What success factors, in your opinion, lead to an employee with a spinal cord or brain injury staying long-term in your company?
- **3. Challenges (15 minutes)**
  - As an employer/supervisor/HR professional, what challenges do you face with a person with a spinal cord or brain injury?
  - Support with topic circle diagram: Are there challenges in these areas?
  - What have been the greatest challenges for you in working with individuals with a spinal cord or brain injury?
  - What are the "red flags" or warning signs for a potential long-term absence of the employee / difficult work situation?
- **4. Support Needs / Offer (10 minutes)**
  - How do you handle difficult situations?
  - How were you supported in these situations, and how would you have liked to be supported?
  - Deepen: By whom, how, type of support, expectations of the support outcome?
  - What responsibility do you see for yourself as an employer? What are you as an employer willing and able to commit/contribute/provide (personally from the perspective of the business) to integrate/employ someone long-term?
  - How can a long-term win-win situation for employers and employees be achieved?
- **5. Closing the Interview (45 minutes)**
  - From your perspective, are there any additions or recommendations you would like to give us/something that we should not forget?
  - Thanks and farewell.
